# Addendum: Genome-wide association study of depression phenotypes in UK Biobank identifies variants in excitatory synaptic pathways

**DOI:** 10.1038/s41467-018-05310-5

**Published:** 2018-08-30

**Authors:** David M. Howard, Mark J. Adams, Masoud Shirali, Toni-Kim Clarke, Riccardo E. Marioni, Gail Davies, Jonathan R. I. Coleman, Clara Alloza, Xueyi Shen, Miruna C. Barbu, Eleanor M. Wigmore, Jude Gibson, Saskia P. Hagenaars, Cathryn M. Lewis, Joey Ward, Daniel J. Smith, Patrick F. Sullivan, Chris S. Haley, Gerome Breen, Ian J. Deary, Andrew M. McIntosh

**Affiliations:** 1 Division of Psychiatry, University of Edinburgh, EH10 5HF Edinburgh, UK; 2 Centre for Cognitive Ageing and Cognitive Epidemiology, University of Edinburgh, EH8 9JZ Edinburgh, UK; 3 Department of Psychology, University of Edinburgh, EH8 9JZ Edinburgh, UK; 4 Social Genetic and Developmental Psychiatry Centre, Institute of Psychiatry, Psychology & Neuroscience, King’s College London, SE5 8AF London, UK; 5 NIHR Biomedical Research Centre for Mental Health, South London and Maudsley NHS Trust, SE5 8AF London, UK; 6 Institute of Health and Wellbeing, University of Glasgow, G12 8RZ Glasgow, UK; 7 Department of Medical Epidemiology and Biostatistics, Karolinska Institutet, 171 77 Stockholm, Sweden; 8 Department of Genetics, University of North Carolina, 27599 Chapel Hill, NC USA; 9 Department of Psychiatry, University of North Carolina, 27599 Chapel Hill, NC USA; 10 Medical Research Council Human Genetics Unit, Institute of Genetics and Molecular Medicine, University of Edinburgh, EH4 2XU Edinburgh, UK

**Keywords:** Genome-wide association studies, Genome-wide association studies, Depression, Depression

Addendum to: *Nature Communications*; 10.1038/s41467-018-03819-3; published online 16 Apr 2018

In this article, a meta-analysis of the significant variants in the UK Biobank sample with a previously published study by 23andMe was included. Due to the individual study results being reported on different scales, the meta-analytic point estimates were not reliable. We have re-analysed the 17 significant variants in the UK Biobank using a comparable approach to 23andMe in Plink. We present an updated version of Table 1 associated with this Addendum that includes the newly calculated comparable effect size estimates for UK Biobank, the updated meta-analysis results and we have extended the table legend to provide information on the updated columns. As a consequence of the new analysis, the original statement in the first paragraph under the subheading “Genome-wide association study of depression” reading “All 17 variants remained significant (*P* < 5 × 10^−8^) in the meta-analysis” should be amended to “10 variants remained significant (*P* < 5 × 10^−8^) in the meta-analysis”. There was no change to the direction of allelic effect in either cohort.

**Table 1:** Independent variants with a genome-wide significant (*P* < 5 × 10^−^^8^) association with broad depression, probable major depressive disorder (MDD), or International Classification of Diseases (ICD)-coded MDD in the UK Biobank

					**UK Biobank**					**23andMe**		**Meta-analysis**		
**Phenotype**	**Chr**	**Marker name**	**Position**	**A1/A2**	**Freq**	**Info**	**log OR (St Err)**	***P*** **-value**	**Gen**e ± 1**0** **kb**	**log OR (St Err)**	***P*** **-value**	**log OR (St Err)**	***P*** **-value**	**Direction**
Broad depression	1	rs10127497	67050144	T/A	0.138	1.00	0.044 (0.0077)	1.26 × 10^−8^	SGIP1	0.0098 (0.0086)	0.257	0.029 (0.0058)	5.70 × 10^−^^7^	++
	1	rs6699744	72825144	T/A	0.612	1.00	0.040 (0.0055)	1.64 × 10^−^^13^	—	0.0328 (0.0064)	2.68 × 10^−^^7^	0.0372 (0.0042)	9.76 × 10^−^^19^	++
	1	rs6424532	73664022	A/G	0.486	1.00	0.029 (0.0053)	3.74 × 10^−^^8^	—	0.0233 (0.006)	1.14 × 10^−^^4^	0.0267 (0.004)	4.10 × 10^−^^11^	++
	1	rs7548151	177026983	A/G	0.084	1.00	0.056 (0.0095)	3.77 × 10^−^^9^	ASTN1	0.006 (0.0104)	0.560	0.0339 (0.0071)	1.97 × 10^−^^6^	++
	5	rs40465	103981726	G/T	0.332	1.00	0.035 (0.0056)	4.37 × 10^−10^	RP11-6N13.1	0.0193 (0.0064)	2.63 × 10^−^^3^	0.0285 (0.0043)	3.53 × 10^−^^11^	++
	6	rs3132685	29945949	A/G	0.130	1.00	−0.059 (0.0081)	2.48 × 10^−^^13^	—	−0.0249 (0.0099)	0.0115	−0.0459 (0.0063)	4.87 × 10^−^^13^	−−
	6	rs112348907	73587953	G/A	0.296	1.00	0.033 (0.0058)	1.44 × 10^−^^8^	—	−0.0004 (0.0067)	0.950	0.019 (0.0045)	2.06 × 10^−^^5^	+−
	7	rs3807865	12250402	A/G	0.412	1.00	0.037 (0.0054)	6.81 × 10^−^^12^	TMEM106B	0.019 (0.0061)	0.002	0.0293 (0.0041)	8.79 × 10^−^^13^	++
	7	rs2402273	117600424	C/T	0.409	1.00	0.032 (0.0054)	1.88 × 10^−^^9^	—	0.0093 (0.0061)	0.130	0.0226 (0.0041)	3.97 × 10^−^^8^	++
	9	rs263575	17033840	A/G	0.460	1.00	−0.030 (0.0053)	2.36 × 10^−^^8^	—	−0.0157 (0.0061)	9.45 × 10^−^^3^	−0.0238 (0.0041)	4.51 × 10^−^^9^	−−
	10	rs1021363	106610839	G/A	0.642	1.00	−0.032 (0.0055)	1.02 × 10^−^^8^	SORCS3	−0.031 (0.0063)	9.34 × 10^−^^7^	−0.0314 (0.0042)	1.04 × 10^−^^13^	−−
	11	rs10501696	88748162	G/A	0.499	0.99	−0.036 (0.0054)	6.42 × 10^−^^11^	GRM5	−0.0251 (0.0066)	1.49 × 10^−^^4^	−0.0315 (0.0043)	1.55 × 10^−^^13^	−−
	13	rs9530139	31847324	T/C	0.195	1.00	−0.040 (0.0067)	2.59 × 10^−^^9^	B3GLCT	−0.0075 (0.0078)	0.338	−0.0265 (0.0052)	2.77 × 10^−^^7^	−−
	15	rs28541419	88945878	G/C	0.231	1.00	−0.035 (0.0064)	2.82 × 10^−^^8^	—	−0.0029 (0.0073)	0.688	−0.0218 (0.0049)	7.75 × 10^−^^6^	−−
Probable MDD	2	rs10929355	15398964	G/T	0.456	1.00	−0.053 (0.0090)	5.89 × 10^−^^9^	NBAS	−0.0078 (0.0061)	0.199	−0.0221 (0.0051)	1.62 × 10^−^^5^	−−
	7	rs5011432	12268668	C/A	0.412	1.00	0.051 (0.0091)	2.10 × 10^−^^8^	TMEM106B	0.022 (0.0061)	3.15 × 10^−^^4^	0.0313 (0.0052)	1.47 × 10^−^^9^	++
ICD-coded MDD	7	rs1554505	1983929	A/G	0.752	1.00	0.114 (0.0191)	2.73 × 10^−^^9^	MAD1L1	0.017 (0.007)	0.015	0.0291 (0.0068)	1.63 × 10^−^^5^	++

The UK Biobank results are from logistic regression analyses in PLINK of variants identified as genome-wide significant using BGENIE. Variants were examined within the 23andMe association analysis of depression^4^ to obtain their reported *P*-values and determine whether their effect was in the same direction as UK Biobank. The allele frequency (Freq) is for the A1 allele within UK Biobank, with the effect size (Log OR) and standard error (St. err.) reported for the A1 allele within UK Biobank, 23andMe and the meta-analysis. The chromosome (Chr) and basepair position is given for the GRCh37 assembly. Imputation accuracy (Info) score of UK Biobank was calculated based on the sample analysed

The fourth sentence in Methods section under the subheading “Replication cohort and meta-analysis” on page 8 was incomplete and should read “Additionally, we used Metal^36^ to conduct an inverse variance-weighted meta-analysis, using LD score regression intercepts^13^ for genomic inflation control.”, adding “using LD score regression intercepts^13^ for genomic inflation control” at the end of the sentence.

We have also included transformed effect sizes and standard errors in our summary statistics deposited on the Edinburgh DataShare website and have updated the doi to 10.7488/ds/2350.

In a second related issue, we used the new BGENIE software package to generate the results with which to conduct gene, region and gene-set analyses to generate data presented in the “Gene and region-based analysis” and “Gene-set pathway analysis” sections, Table 2, Supplementary Table 6, Supplementary Data 6–12, and Supplementary Figures 7–9. BGENIE reported minor allele frequencies (MAF) across the whole of UK Biobank (*n* = 487,409) rather than based on those individuals that were included in each of the association analyses (broad depression *n* = 322,580; probable major depressive disorder *n* = 174,519; International Classification of Diseases-coded major depressive disorder *n* = 217,584). Therefore the results reported in the “Gene and region-based analysis” and “Gene-set pathway analysis” were based on the MAF across the whole of UK Biobank as opposed to what we described in the methods section. To correct these errors in our initial analysis, we have now re-analysed the data using the MAF based on only those individuals included in the respective association analysis. This has resulted in 73 rather than 78 significant genes for broad depression, three rather than two genes for probable MDD, and zero genes rather than one gene for ICD-coded MDD which was originally described in the “Gene and region-based analysis” section in the results. The gene-based results descri bed on page 3 under the subheading “Gene and region-based analyses” should read “We used the MAGMA^18^ package to identify genes with a significant effect (*P* < 2.77 × 10^−6^) on each phenotype. There were 73 genes significantly associated with broad depression (Supplementary Data 6, associated with this Addendum), and three genes that were associated with probable MDD (Supplementary Data 7, associated with this Addendum)”. Updated versions of Supplementary Note 1, Supplementary Figures 7, 8, and 9 in the Supplementary Information file, and Supplementary Data 6 and 7 are included here to reflect these changes in the data. The original Supplementary Data 8 should be disregarded as with the new analysis no genome-wide significant SNPs for ICD-coded MDD are detected. In the region-based analysis described on page 3 number of regions assessed increased from 8308 to 8345 requiring a slight change in the significance threshold from 6.02 × 10^−8^ to 5.99 × 10^−6^ so that the sentence in the second paragraph under the subheading “Gene and region-based analyses” now reads “We also used MAGMA to identify genomic regions, defined by recombination hotspots, with a statistically significant effect (*P* < 5.99 × 10^−6^) on each phenotype”. The number of significant regions remained the same and updated estimates of effect sizes of regions may be found in the updated Supplementary Data 8, 10, and 11 associated with this Addendum. The significant gene-sets described under the subheading “Gene-set pathway analysis” remained the same with slight differences in number of genes in each pathway, effect sizes and *p*-values. The first sentence in this section now reads: “We conducted gene-set enrichment analysis^19,^^20^ and identified five significant pathways for broad depression after applying correction for multiple testing; GO_EXCITATORY_SYNAPSE (beta = 0.342 ± 0.069, *P*_corrected _= 0.003), GO_MECHANOSENSORY_BEHAVIOR (beta = 1.270 ± 0.294, *P*_corrected _= 0.047), GO_POSTSYNAPSE (beta = 0.248 ± 0.050, *P*_corrected_ = 0.003), GO_NEURON_SPINE (beta = 0.390 ± 0.089, *P*_corrected_ = 0.019) and GO_DENDRITE (beta = 0.200 ± 0.044, P_corrected_ = 0.021) (Table 2)”. Amended versions of Table 2, Supplementary Data 12 and Supplementary Table 6 in the Supplementary Information file are included in this Addendum.

**Table 2:** Pathways with a significant effect (*P*_corrected_ < 0.05) on broad depression following multiple testing correction identified through gene-set enrichment analysis

**Phenotype**	**Pathway**	**Number of genes**	**Beta (St. Err.)**	***P*** **-value**	***P***_**Corrected**_
Broad depression	GO_EXCITATORY_SYNAPSE	184	0.342 (0.069)	3.45 × 10^−^^7^	0.003
	GO_POSTSYNAPSE	354	0.248 (0.050)	3.88 × 10^−^^7^	0.003
	GO_NEURON_SPINE	115	0.390 (0.089)	2.72 × 10^−^^6^	0.019
	GO_DENDRITE	425	0.200 (0.044)	3.08 × 10^−^^6^	0.021
	GO_MECHANOSENSORY_BEHAVIOR	12	1.270 (0.294)	8.04 × 10^−^^6^	0.047

The described errors have not been fixed in the original article. We reaffirm that the changes do not change the main conclusions of the manuscript.

## Electronic supplementary material

UKBiobank GWAS of Depression Supplementary Info

SupplementaryData6

SupplementaryData7

SupplementaryData9

SupplementaryData10

SupplementaryData11

SupplementaryData12
