# Author Correction: Genome-wide association study of depression phenotypes in UK Biobank identifies variants in excitatory synaptic pathways

**DOI:** 10.1038/s41467-021-22411-w

**Published:** 2021-03-25

**Authors:** David M. Howard, Mark J. Adams, Masoud Shirali, Toni-Kim Clarke, Riccardo E. Marioni, Gail Davies, Jonathan R. I. Coleman, Clara Alloza, Xueyi Shen, Miruna C. Barbu, Eleanor M. Wigmore, Jude Gibson, 23andMe Research Team, Michelle Agee, Babak Alipanahi, Adam Auton, Robert K. Bell, Katarzyna Bryc, Sarah L. Elson, Pierre Fontanillas, Nicholas A. Furlotte, David A. Hinds, Karen E. Huber, Aaron Kleinman, Nadia K. Litterman, Jennifer C. McCreight, Matthew H. McIntyre, Joanna L. Mountain, Elizabeth S. Noblin, Carrie A. M. Northover, Steven J. Pitts, J. Fah Sathirapongsasuti, Olga V. Sazonova, Janie F. Shelton, Suyash Shringarpure, Chao Tian, Joyce Y. Tung, Vladimir Vacic, Catherine H. Wilson, Saskia P. Hagenaars, Cathryn M. Lewis, Joey Ward, Daniel J. Smith, Patrick F. Sullivan, Chris S. Haley, Gerome Breen, Ian J. Deary, Andrew M. McIntosh

**Affiliations:** 1 Division of Psychiatry, University of Edinburgh, Edinburgh, EH10 5HF UK; 2 Centre for Cognitive Ageing and Cognitive Epidemiology, University of Edinburgh, Edinburgh, EH8 9JZ UK; 3 Department of Psychology, University of Edinburgh, Edinburgh, EH8 9JZ UK; 4 Social Genetic and Developmental Psychiatry Centre, Institute of Psychiatry, Psychology & Neuroscience, King’s College London, London, SE5 8AF UK; 5 NIHR Biomedical Research Centre for Mental Health, South London and Maudsley NHS Trust, London, SE5 8AF UK; 6 Institute of Health and Wellbeing, University of Glasgow, Glasgow, G12 8RZ UK; 7 Department of Medical Epidemiology and Biostatistics, Karolinska Institutet, Stockholm, 171 77 Sweden; 8 Department of Genetics, University of North Carolina, Chapel Hill, 27599 NC USA; 9 Department of Psychiatry, University of North Carolina, Chapel Hill, 27599 NC USA; 10 Medical Research Council Human Genetics Unit, Institute of Genetics and Molecular Medicine, University of Edinburgh, Edinburgh, EH4 2XU UK; 11 23andMe, Inc., Mountain View, CA 94041 USA

Correction to: *Nature Communications* 10.1038/s41467-018-03819-3, published online 16 April 2018.

The original version of this Article contained an error in the ‘Methods’ subsection entitled ‘Broad depression phenotype’, which incorrectly read ‘“Have you ever seen a psychiatrist for nerves, anxiety, tension or depression”? (UK Biobank field 2010)’. The same error was made three times in the subsections ‘Probable MDD phenotype’ and ‘ICD-coded MDD phenotype’, which read ‘(UK Biobank fields: 2090 and 2010)’. The correct version states ‘2100’ in place of ‘2010’ in each case. This has been corrected in both the PDF and HTML versions of the Article.

