# Supplementary material for: Genome-wide association study of depression phenotypes in UK Biobank identifies variants in excitatory synaptic pathways
Source: Nat Commun. 2018 Apr 16;9:1470. doi: 10.1038/s41467-018-03819-3 (PMC5902628; doi:10.1038/s41467-018-03819-3)
Supplement: Supplementary file 3 — Description of Additional Supplementary Files [file 41467_2018_3819_MOESM3_ESM.pdf]

Description of Additional Supplementary Files

File Name: Supplementary Data 1
Description: Independent variants with a genome-wide significant (P < 5 × 10-8) association with either broad depression, probable major depressive disorder (MDD), or International Classification of Diseases-coded MDD in UK Biobank. Effect size (beta), standard error and p-values are the results from the linear model across the three phenotypes examined. Odds Ratios are calculated using a logistic regression model. Cells surrounded by a border indicate the phenotype for which statistical significance was initially observed.

File Name: Supplementary Data 2
Description: Variants with a P-value < 1 × 10-6 for an association with broad depression.

File Name: Supplementary Data 3
Description: Variants with a P-value < 1 × 10-6 for an association with probable MDD

File Name: Supplementary Data 4
Description: Variants with a P-value < 1 × 10-6 for an association with ICD-coded MDD

File Name: Supplementary Data 5
Description: Genetic correlations (rg) between the three depression-related phenotypes and 235 other behavioural and disease related traits, sorted by significance with broad depression.

File Name: Supplementary Data 6
Description: Genome-wide significant gene-based hits (P < 2.77 x 10-6) in the MAGMA gene-based analysis for broad depression. NSNPS is the number of SNPs in the gene; NiSNPs is the number of independent SNPs in the gene.

File Name: Supplementary Data 7
Description: Genome-wide significant gene-based hits (P < 2.77 x 10-6) in the MAGMA gene-based analysis for probable MDD. NSNPS is the number of SNPs in the gene; NiSNPs is the number of independent SNPs in the gene.

File Name: Supplementary Data 8
Description: Genome-wide significant gene-based hits (P < 2.77 x 10-6) in the MAGMA gene-based analysis for ICD-coded MDD. NSNPS is the number of SNPs in the gene; NiSNPs is the number of independent SNPs in the gene.

File Name: Supplementary Data 9
Description: Genome-wide significant region-based hits (P < 6.02 x 10-6) in the MAGMA region-based analysis for broad depression. NSNPS is the number of SNPs in the gene; NiSNPs is the number of independent SNPs in the gene. RegNum is the region number by clustering nearby significant blocks by at less than 100K bp distance together as a region.

File Name: Supplementary Data 10
Description: Genome-wide significant region-based hits (P < 6.02 x 10-6) in the MAGMA region-based analysis for probable MDD. NSNPS is the number of SNPs in the gene; NiSNPs is the number of independent SNPs in the gene. RegNum is the region number by clustering nearby significant blocks by at less than 100K bp distance together as a region.

File Name: Supplementary Data 11
Description: Genome-wide significant region-based hits (P < 6.02 x 10-6) in the MAGMA region-based analysis for ICD-coded MDD . NSNPS is the number of SNPs in the gene; NiSNPs is the number of independent SNPs in the gene. RegNum is the region number by clustering nearby significant blocks by at less than 100K bp distance together as a region.

File Name: Supplementary Data 12
Description: Genes located within the five significant gene-sets identified for broad depression. P-values are those obtained from MAGMA for each genes association with broad depression along with an indication of significance

File Name: Supplementary Data 13
Description: Enrichment across 209 tissues for each phenotype using DEPICT.

File Name: Supplementary Data 14
Description: Expression quantitative trait loci identified by analysis of significant variants using the GTEx online portal and the significance of their expression in the specified tissues.
