# Supplementary material for: Addendum: Genome-wide association study of depression phenotypes in UK Biobank identifies variants in excitatory synaptic pathways
Source: Nat Commun. 2018 Aug 30;9:3578. doi: 10.1038/s41467-018-05310-5 (PMC6117285; doi:10.1038/s41467-018-05310-5)
Supplement: Supplementary file 1 — UKBiobank GWAS of Depression Supplementary Info [file 41467_2018_5310_MOESM1_ESM.pdf]

Genome-wide association study of depression phenotypes in UK Biobank identifies variants in excitatory synaptic pathways

Howard et al.

Supplementary Note 1

Extended discussion of protein coding genes

A genome-wide significant variant associated with broad depression and identified as an eQTL by GTEx on chromosome 1 (rs6699744; 72,825,144 bp; $P = 1.64 \times 10^{-13}$), was close to another significant variant (rs11209948; 72,811,904 bp; $P = 8.38 \times 10^{-11}$) associated with MDD within the Hyde, et al. [1] study. Both of these variants were close to the Neural Growth Regulator 1 (*NEGR1*) gene which was associated with MDD in the Major Depressive Disorder Working Group of the Psychiatric Genomics Consortium., et al. [2] study. Another significant variant for broad depression on chromosome 1 (rs7548151; 177,026,983 bp; $P = 3.87 \times 10^{-9}$) was within 30 Kb of the microRNA 488 (*MIR488*) coding region. MicroRNAs are involved in post-transcriptional regulation of gene expression affecting both stability and translation of mRNAs; MIR488 is enriched in brain, and transcriptome analysis has demonstrated that altered expression of MIR488 is nominally associated with stress response and panic disorder[3].

A significant variant (rs1021363; 106,610,839 bp; $P = 1.04 \times 10^{-8}$; Supplementary Figure 5) on chromosome 10 was associated with broad depression in our study and was within 4 Kb of another variant (rs10786831; 106,614,571 bp; $P = 8.11 \times 10^{-9}$) found to be associated within the Hyde, et al. [1] MDD study, and close to a variant (rs61867293; 106,563,924 bp; $P = 7.0 \times 10^{-10}$) associated with MDD in the Major Depressive Disorder Working Group of the Psychiatric Genomics Consortium., et al. [2] study. These variants are close to the sortilin related VPS10 domain containing receptor 3 (*SORCS3*) protein coding gene, which is associated with Alzheimer's disease risk and implicated in processing of amyloid precursor protein processing[4]. *SORCS3* is also expressed at high levels in the brain and may influence glutamate receptor trafficking[5].

There were up to eight variants in the gene-rich MHC region that could have been classified as independent; however, the complexity of the genetic architecture across this region may confound this interpretation. Therefore, we report only the most significant variant (rs3132685; 29,945,949 bp; $P = 2.47 \times 10^{-13}$). The MHC region has been associated with both schizophrenia and bipolar disorder

across multiple studies[6-8], as well as an early-onset and recurrent form of depression[9]. A closer examination of the MHC region with respect to psychiatric disorders is warranted, based on previous studies and our findings.

There was a genome-wide significant variant (rs10501696; 88,748,162 bp; $P = 6.73 \times 10^{-11}$; Supplementary Figure 6) located on chromosome 11 that overlapped the glutamate metabotropic receptor 5 (*GRM5*) protein coding gene. *GRM5* is expressed in the brain and facilitates glutamatergic neurotransmission. *GRM5* has previously been associated with a range of behavioural and neurological phenotypes such as depression[10], OCD[11], epilepsy[12], smoking[13], Alzheimer's disease[14,15], autism[16], and schizophrenia[17]. A recent study found a role for Metabotropic glutamate receptor 5 (*mGluR5*) in relation to stress-induced depression in mice[18], and *GRM5* antagonists have been shown to have anxiolytic and anti-depressant properties[19,20].

A variant on chromosome 7 (rs1554505; 1,983,929 bp; $P = 2.74 \times 10^{-9}$) was associated with ICD-coded MDD. This variant is located in the MAD1 mitotic arrest deficient-like 1 (*MAD1L1*) gene coding region. *MAD1L1* is a known susceptibility locus for schizophrenia[21-23], and a recent study found differential reward processing during an fMRI task in carriers of a *MAD1L1* bipolar risk allele[24].

Gene-based and region-based analyses

Additional analyses were conducted in order to assess the simultaneous effect of multiple genetic variants on specific genes and within specific regions. The gene-based analysis identified a total of 75 genome-wide significant ($P < 2.77 \times 10^{-6}$) genes across the three phenotypes. The transmembrane protein 106B (*TMEM106B*) gene coding region (Supplementary Figure 4) was identified in both the broad depression and probable MDD. *TMEM106B* encodes a type II transmembrane protein of unknown function, although it represents a risk factor for Frontotemporal lobar degeneration, especially in patients with progranulin mutations[25]. An inverse relationship between *TMEM106B* (downregulation) and progranulin (upregulation) expression levels in Alzheimer's disease brains has

been reported that demonstrated the role of *TMEM106B* gene in the pathological processes of Alzheimer's disease[26].

Our region-based analysis identified 59 genome-wide significant ($P < 5.99 \times 10^{-6}$) regions across the three phenotypes, with three of these regions detected in more than one phenotype. The region-based method detected regions harbouring several known genes that are reported to have effect on depression and other mental diseases that were not detected in our gene-based analysis.

The region-based analysis of both broad depression and probable MDD detected a significant region on chromosome 1 that contained the glutamate ionotropic receptor kainate type subunit 3 (*GRIK3*) protein coding region. Glutamate receptors are the main excitatory neurotransmitter receptors in the mammalian brain. Moreover, these receptors are active in several neurophysiologic processes. *GRIK3* has been associated with schizophrenia[27,28], neuroticism[29] and recurrent MDD[30,31]. Higher levels of *GRIK3* have been reported in MDD suicides compared to MDD non-suicides, with *GRIK3* expression being a strong predictor of suicide[32].

The analysis of broad depression and ICD-coded MDD both detected a significant region containing the Receptor tyrosine-protein kinase erbB-4X (*ERBB4*) protein coding region. *ERBB4* is a member of the Tyr protein kinase family and the epidermal growth factor receptor subfamily. *ERBB4* has been previously linked to schizophrenia[33], and impairments in the link between Neuregulin 1 (*NRG1*) and *ERBB4* signalling are associated with schizophrenia[34] and anxiety behaviours[35].

The broad depression analysis identified the dihydropyrimidine dehydrogenase (*DPYD*) gene coding region, which has been associated with schizophrenia, bipolar disorder[23], and borderline personality disorder[36]. Another region contained the Neurexin 1 (*NRXN1*) gene coding region, which has been associated with Tourette syndrome[37] and non-syndromic autism spectrum disorder[38]. A further region contained the regulator of G protein signalling 6 (*RGS6*) coding region, which has been previously associated with alcoholism[39], depression, anxiety[40], and Parkinson's disease[41].

References

1. Hyde, C.L. *et al.* Identification of 15 genetic loci associated with risk of major depression in individuals of European descent. *Nature Genetics* **48**, 1031-1036 (2016).
2. Major Depressive Disorder Working Group of the Psychiatric Genomics Consortium., Wray, N.R. & Sullivan, P.F. Genome-wide association analyses identify 44 risk variants and refine the genetic architecture of major depression. *bioRxiv* (2017).
3. Muiños-Gimeno, M. *et al.* Human microRNAs miR-22, miR-138-2, miR-148a, and miR-488 Are Associated with Panic Disorder and Regulate Several Anxiety Candidate Genes and Related Pathways. *Biological Psychiatry* **69**, 526-533 (2011).
4. Reitz, C. *et al.* Independent and epistatic effects of variants in VPS10-d receptors on Alzheimer disease risk and processing of the amyloid precursor protein (APP). *Translational Psychiatry* **3**, e256 (2013).
5. Breiderhoff, T. *et al.* Sortilin-related receptor SORCS3 is a postsynaptic modulator of synaptic depression and fear extinction. *PLOS ONE* **8**, e75006 (2013).
6. Bergen, S.E. *et al.* Genome-wide association study in a Swedish population yields support for greater CNV and MHC involvement in schizophrenia compared with bipolar disorder. *Molecular Psychiatry* **17**, 880-886 (2012).
7. Ruderfer, D.M. *et al.* Polygenic dissection of diagnosis and clinical dimensions of bipolar disorder and schizophrenia. *Molecular Psychiatry* **19**, 1017-1024 (2014).
8. Sleiman, P. *et al.* GWAS meta analysis identifies TSNARE1 as a novel Schizophrenia / Bipolar susceptibility locus. *Scientific Reports* **3**, 3075 (2013).
9. Shyn, S.I. *et al.* Novel loci for major depression identified by genome-wide association study of Sequenced Treatment Alternatives to Relieve Depression and meta-analysis of three studies. *Molecular Psychiatry* **16**, 202-215 (2011).
10. Chandley, M.J. *et al.* Elevated gene expression of glutamate receptors in noradrenergic neurons from the locus coeruleus in major depression. *International Journal of Neuropsychopharmacology* **17**, 1569-1578 (2014).
11. Akkus, F. *et al.* Metabotropic glutamate receptor 5 binding in patients with obsessive-compulsive disorder. *International Journal of Neuropsychopharmacology* **17**, 1915-1922 (2014).
12. Kandratavicius, L. *et al.* Distinct increased metabotropic glutamate receptor type 5 (mGluR5) in temporal lobe epilepsy with and without hippocampal sclerosis. *Hippocampus* **23**, 1212-1230 (2013).
13. Hulka, L.M. *et al.* Smoking but not cocaine use is associated with lower cerebral metabotropic glutamate receptor 5 density in humans. *Mol Psychiatry* **19**, 625-632 (2014).
14. Tsamis, K.I., Mytilinaios, D.G., Njau, S.N. & Baloyannis, S.J. Glutamate receptors in human caudate nucleus in normal aging and Alzheimer's disease. *Current Alzheimer Research* **10**, 469-475 (2013).
15. Haas, L.T. *et al.* Metabotropic glutamate receptor 5 couples cellular prion protein to intracellular signalling in Alzheimer's disease. *Brain* **139**, 526-546 (2016).
16. Fatemi, S.H., Folsom, T.D., Kneeland, R.E. & Liesch, S.B. Metabotropic glutamate receptor 5 upregulation in children with autism is associated with underexpression of both Fragile X mental retardation protein and GABA(A) receptor beta 3 in adults with autism. *Anatomical Record* **294**, 10.1002/ar.21299 (2011).
17. Matosin, N. *et al.* Alterations of mGluR5 and its endogenous regulators Norbin, Tamalin and Preso1 in schizophrenia: towards a model of mGluR5 dysregulation. *Acta Neuropathologica* **130**, 119-129 (2015).
18. Shin, S. *et al.* mGluR5 in the nucleus accumbens is critical for promoting resilience to chronic stress. *Nature Neuroscience* **18**, 1017-1024 (2015).

19. Tatarczyńska, E. *et al.* Potential anxiolytic- and antidepressant-like effects of MPEP, a potent, selective and systemically active mGlu5 receptor antagonist. *British Journal of Pharmacology* **132**, 1423-1430 (2001).
20. Spooren, W., Lesage, A., Lavreysen, H., Gasparini, F. & Steckler, T. Metabotropic glutamate receptors: their therapeutic potential in anxiety. in *Behavioral Neurobiology of Anxiety and Its Treatment* (eds. Stein, M.B. & Steckler, T.) 391-413 (Springer Berlin Heidelberg, Berlin, Heidelberg, 2010).
21. O'Dushlaine, C. *et al.* Molecular pathways involved in neuronal cell adhesion and membrane scaffolding contribute to schizophrenia and bipolar disorder susceptibility. *Molecular Psychiatry* **16**, 286-292 (2011).
22. Su, L. *et al.* Genetic association of GWAS-supported MAD1L1 gene polymorphism rs12666575 with schizophrenia susceptibility in a Chinese population. *Neuroscience Letters* **610**, 98-103 (2016).
23. Ripke, S. *et al.* Genome-wide association analysis identifies 13 new risk loci for schizophrenia. *Nature Genetics* **45**, 1150-9 (2013).
24. Trost, S. *et al.* Investigating the impact of a genome-wide supported bipolar risk variant of MAD1L1 on the human reward system. *Neuropsychopharmacology* **41**, 2679-2687 (2016).
25. Cruchaga, C. *et al.* TMEM106B gene polymorphism is associated with age at onset in granulin mutation carriers and plasma granulin protein levels. *Archives of neurology* **68**, 581-586 (2011).
26. Satoh, J.-i. *et al.* TMEM106B expression is reduced in Alzheimer’s disease brains. *Alzheimer's Research & Therapy* **6**, 17 (2014).
27. Djurovic, S. *et al.* A possible association between schizophrenia and GRIK3 polymorphisms in a multicenter sample of Scandinavian origin (SCOPE). *Schizophrenia Research* **107**, 242-248 (2009).
28. Greenwood, T.A. *et al.* Genetic assessment of additional endophenotypes from the Consortium on the Genetics of Schizophrenia Family Study. *Schizophrenia Research* **170**, 30-40 (2016).
29. Smith, D.J. *et al.* Genome-wide analysis of over 106 000 individuals identifies 9 neuroticism-associated loci. *Molecular Psychiatry* **21**, 749-757 (2016).
30. Luciano, M. *et al.* Association of existing and new candidate genes for anxiety, depression and personality traits in older people. *Behavior Genetics* **40**, 518-532 (2010).
31. Schiffer, H.H. & Heinemann, S.F. Association of the human kainate receptor GluR7 gene (GRIK3) with recurrent major depressive disorder. *American Journal of Medical Genetics Part B: Neuropsychiatric Genetics* **144B**, 20-26 (2007).
32. Gray, A.L., Hyde, T.M., Deep-Soboslay, A., Kleinman, J.E. & Sodhi, M.S. Sex differences in glutamate receptor gene expression in major depression and suicide. *Molecular Psychiatry* **20**, 1057-1068 (2015).
33. Law, A.J., Kleinman, J.E., Weinberger, D.R. & Weickert, C.S. Disease-associated intronic variants in the ErbB4 gene are related to altered ErbB4 splice-variant expression in the brain in schizophrenia. *Human Molecular Genetics* **16**, 129-141 (2007).
34. Li, B., Woo, R.-S., Mei, L. & Malinow, R. The neuregulin-1 receptor ErbB4 controls glutamatergic synapse maturation and plasticity. *Neuron* **54**, 583-597 (2007).
35. Bi, L.-L. *et al.* Amygdala NRG1-ErbB4 is critical for the modulation of anxiety-like behaviors. *Neuropsychopharmacology* **40**, 974-986 (2015).
36. Witt, S.H. *et al.* Genome-wide association study of borderline personality disorder reveals genetic overlap with bipolar disorder, major depression and schizophrenia. *Translational Psychiatry* **7**, e1155 (2017).
37. Huang, A.Y. *et al.* Rare Copy Number Variants in NRXN1 and CNTN6 Increase Risk for Tourette Syndrome. *Neuron* **94**, 1101-1111.e7 (2017).
38. Onay, H. *et al.* Mutation analysis of the NRXN1 gene in autism spectrum disorders. *Balkan Journal of Medical Genetics* **19**, 17-22 (2016).
39. Stewart, A. *et al.* Regulator of G protein signaling 6 is a critical mediator of both reward-related behavioral and pathological responses to alcohol. *Proc Natl Acad Sci U S A* **112**, E786-95 (2015).

40. Stewart, A. *et al.* Regulator of G-protein signaling 6 (RGS6) promotes anxiety and depression by attenuating serotonin-mediated activation of the 5-HT(1A) receptor-adenylyl cyclase axis. *FASEB J* **28**, 1735-44 (2014).
41. Bifsha, P., Yang, J., Fisher, R.A. & Drouin, J. Rgs6 is required for adult maintenance of dopaminergic neurons in the ventral substantia nigra. *PLoS Genetics* **10**, e1004863 (2014).

Supplementary Note 2

Phenotype Definitions

Broad depression definition

- **Cases**
    - From touchscreen questionnaire at recruitment:
        - "Have you ever seen a GP/psychiatrist for nerves, anxiety, tension or depression?" (Fields 2090 and 2100) **- Yes**
    - From Hospital Episodes Data from UK bodies (English HES Data, Scottish Morbidity Register, Patient Episode Data) (Fields 41202 and 41204)
        - Any primary or secondary diagnosis of ICD-10 Codes for mood disorders
            - F32 - Single Episode Depression
            - F33 - Recurrent Depression
            - F34 - Persistent mood disorders (Cyclothymia, Dysthymia)
            - F38 - Other mood disorders
            - F39 - Unspecified mood disorders
- **Controls**
    - From touchscreen questionnaire at recruitment:
        - "Have you ever seen a GP/psychiatrist for nerves, anxiety, tension or depression?" (Fields 2090 and 2100) **- No**

Probable major depressive disorder (MDD) definition

- **Cases**
    - From touchscreen questionnaire at recruitment:
        - "Have you ever seen a GP/psychiatrist for nerves, anxiety, tension or depression?" (Fields 2090 and 2100) **- Yes**
            - **EITHER** "Looking back over your life, have you ever had a time when you were feeling depressed or down for at least a whole week?" (Field 4598) - **Yes**
                - "How many weeks was the longest period when you were feeling depressed or down?" (Field 4609) **- More than 2**
            - **OR** "Have you ever had a time when you were uninterested in things or unable to enjoy the things you used to for at least a whole week?" (Field 4631) **- Yes**
                - "How many weeks was the longest period when you were uninterested in things or unable to enjoy the things you used to?" (Field 5375)**- More than 2**
    - From Hospital Episodes Data from UK bodies (English HES Data, Scottish Morbidity Register, Patient Episode Data) (Fields 41202 and 41204)

- Any primary or secondary diagnosis of ICD-10 Codes for mood disorders
    - F32 - Single Episode Depression
    - F33 - Recurrent Depression
    - F34 - Persistent mood disorders (Cyclothymia, Dysthymia)
    - F38 - Other mood disorders
    - F39 - Unspecified mood disorders

- **Controls**
  - From touchscreen questionnaire at recruitment:
    - "Have you ever seen a GP/psychiatrist for nerves, anxiety, tension or depression?" (Fields 2090 and 2100) **- No**
    - "Looking back over your life, have you ever had a time when you were feeling depressed or down for at least a whole week?" (Field 4598) **- No**
    - "Have you ever had a time when you were uninterested in things or unable to enjoy the things you used to for at least a whole week?" Field 4631) **- No**

ICD-coded MDD definition

- **Cases**
  - From Hospital Episodes Data from UK bodies (English HES Data, Scottish Morbidity Register, Patient Episode Data) (Fields 41202 and 41204)
    - Any primary or secondary diagnosis of ICD-10 Codes for mood disorders
      - F32 - Single Episode Depression
      - F33 - Recurrent Depression
      - F34 - Persistent mood disorders (Cyclothymia, Dysthymia)
      - F38 - Other mood disorders
      - F39 - Unspecified mood disorders
- **Controls**
  - **NOT** a case per the Smith definition using touchscreen information (above)
  - From Hospital Episodes Data from UK bodies (English HES Data, Scottish Morbidity Register, Patient Episode Data) (Fields 41202 and 41204)
    - No primary or secondary diagnosis of ICD-10 Codes for mood disorders, as above

Exclusions applied to all definitions

- **For cases and controls**
  - Bipolar (ICD codes F30, F31 or non-cancer illness code 1291)
  - Multiple personality disorder (ICD code F44.8)
  - Schizophrenia / psychosis (ICD codes F2*, or non-cancer illness code 1289)
  - Treatment/medication codes for antipsychotics (Field 20003):

- abilify 5mg tablet 1141202024
- amisulpride 1141153490
- aripiprazole 1141195974
- benperidol 1140867078
- camcolit 250 tablet 1140867494
- carbagen sr 200mg m/r tablet 1141171566
- carbamazepine 2038459704
- carbamazepine product 1140872064
- chlorpromazine 1140879658
- clopixol 2mg tablet 1140867342
- clozapine 1140867420
- clozaril 25mg tablet 1140882320
- convulex 150mg e/c capsule 1140872216
- cpz - chlorpromazine 1140910358
- denzapine 25mg tablet 1141200458
- depakote 250mg e/c tablet 1141172838
- dolmatil 200mg tablet 1140867306
- dozic 1mg/ml oral liquid 1140867180
- epilim 100mg crushable tablet 1140872200
- fentazin 2mg tablet 1140867210
- fluphenazine decanoate 1140867398
- fluphenazine 1140882098
- haldol 5mg tablet 1140867184
- haloperidol 1140867168
- largactil 10mg tablet 1140863416
- levomepromazine 1140909802
- liskonum 450mg m/r tablet 1140867498
- lithium product 1140867490
- lithonate 400mg m/r tablet 1140910976
- methotrimeprazine 1140867118
- modecate 12.5mg/0.5ml oily injection 1140867456
- olanzapine 1140928916
- orlept 200mg e/c tablet 1140872268
- pericyazine 1140867134
- perphenazine 1140867208
- pimozide 1140867218
- piportil depot 50mg/1ml oily injection 1140867572
- pipothiazine 1140879674
- pipotiazine 1140909804
- priadel 200mg m/r tablet 1140867504
- prochlorperazine 1140868170
- promazine 1140879746
- quetiapine 1141152848
- risperdal 0.5mg tablet 1141177762
- risperidone 1140867444
- serenace 500micrograms capsule 1140867092
- seroquel 25mg tablet 1141152860
- sodium valproate 1140872198
- stelazine 1mg tablet 1140867244
- stemetil 5mg tablet 1140868172
- sulpiride 1140867304

        - tegretol 100mg tablet 1140872072
        - thioridazine 1140879750
        - trifluoperazine 1140868120
        - valproic acid 1140872214
        - zaponex 25mg tablet 1141201792
        - zuclopenthixol 1140882100
        - zyprexa 2.5mg tablet 1141167976

- **For controls**
    - Treatment/medication codes for antidepressants (Field 20003):
        - allegron 10mg tablet 1140867820
        - amitriptyline hydrochloride+perphenazine 10mg/2mg tablet 1140867948
        - amitriptyline 1140879616
        - amitriptyline+chlordiazepoxide 12.5mg/5mg capsule 1140867938
        - anafranil 10mg capsule 1140867690
        - cipralex 5mg tablet 1141190158
        - cipramil 10mg tablet 1141151946
        - citalopram 1140921600
        - clomipramine 1140879620
        - cymbalta 30mg gastro-resistant capsule 1141201834
        - depixol 3mg tablet 1140867152
        - dosulepin 1140909806
        - dothiepin 1140879628
        - doxepin1140867640
        - duloxetine 1141200564
        - edronax 4mg tablet 1141151982
        - efexor 37.5mg tablet 1140916288
        - escitalopram 1141180212
        - faverin 50mg tablet 1140867860
        - fluanxol 500micrograms tablet 1140867952
        - fluoxetine 1140879540
        - flupenthixol 1140867150
        - flupentixol 1140909800
        - fluphenazine hydrochloride+nortriptyline 1.5mg/30mg tablet 1140867940
        - fluvoxamine 1140879544
        - imipramine 1140879630
        - isocarboxazid 1140867856
        - lofepramine 1140867726
        - lustral 50mg tablet 1140867884
        - manerix 150mg tablet 1140867922
        - maoi - tranylcypromine 1140910820
        - mianserin 1140879556
        - mirtazapine 1141152732
        - moclobemide 1140867920
        - molipaxin 50mg capsule 1140882244
        - nardil 15mg tablet 1140867852
        - nortriptyline 1140867818
        - oxactin 20mg capsule 1141174756
        - parnate 10mg tablet 1140867916
        - paroxetine 1140867888
        - phenelzine 1140867850

- prothiaden 25mg capsule 1140867624
- prozac 20mg capsule 1140867876
- reboxetine 1141151978
- seroxat 20mg tablet 1140882236
- sertraline 1140867878
- st john's wort/hypericum [ctsu] 1201
- sinequan 10mg capsule 1140882312
- surmontil 10mg tablet 1140867758
- tofranil 10mg tablet 1140867712
- tranylcypromine 1140867914
- tranylcypromine+trifluoperazine 10mg/1mg tablet 1140867944
- trazodone 1140879634
- trimipramine 1140867756
- triptafen tablet 1140867934
- tryptophan product 1140867960
- venlafaxine 1140916282
- yentreve 20mg gastro-resistant capsule 1141200570
- zispin 30mg tablet 1141152736

- Mood disorders (ICD codes F32, F33, F34, F38, F39 or non-cancer illness code 1286)

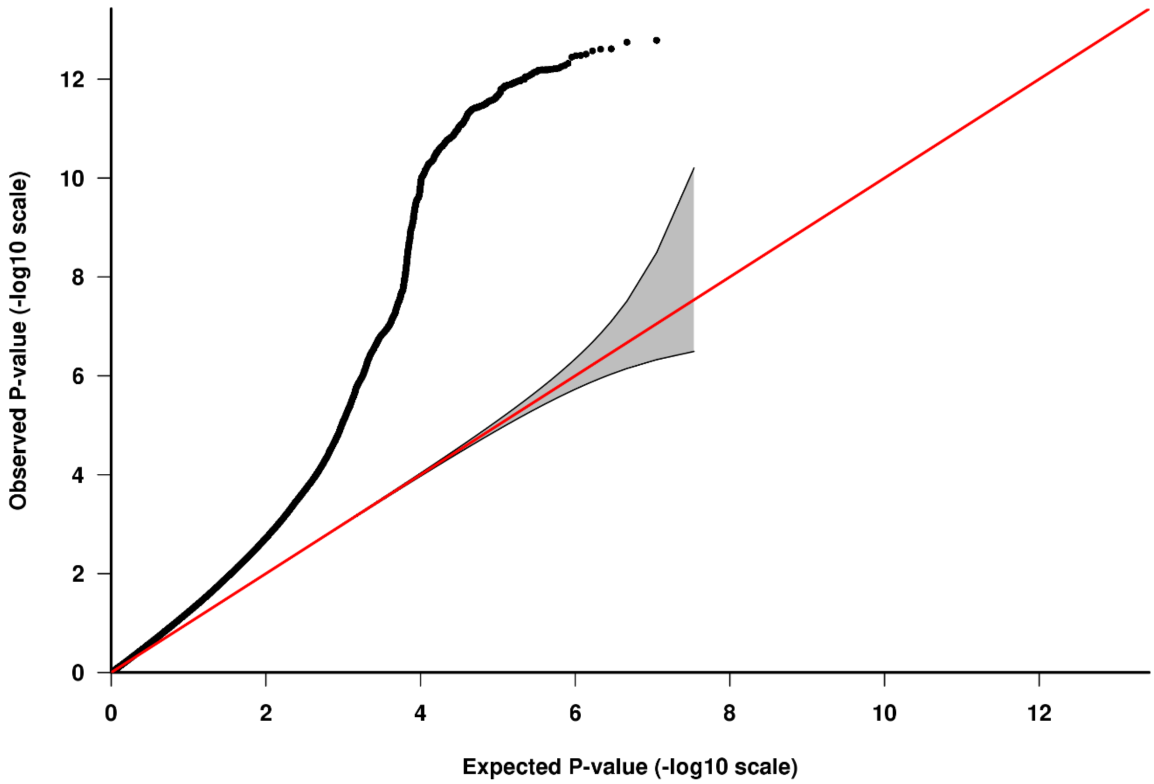

Supplementary Figure 1. Q-Q plot of the observed *P*-values for an association with broad depression on that expected

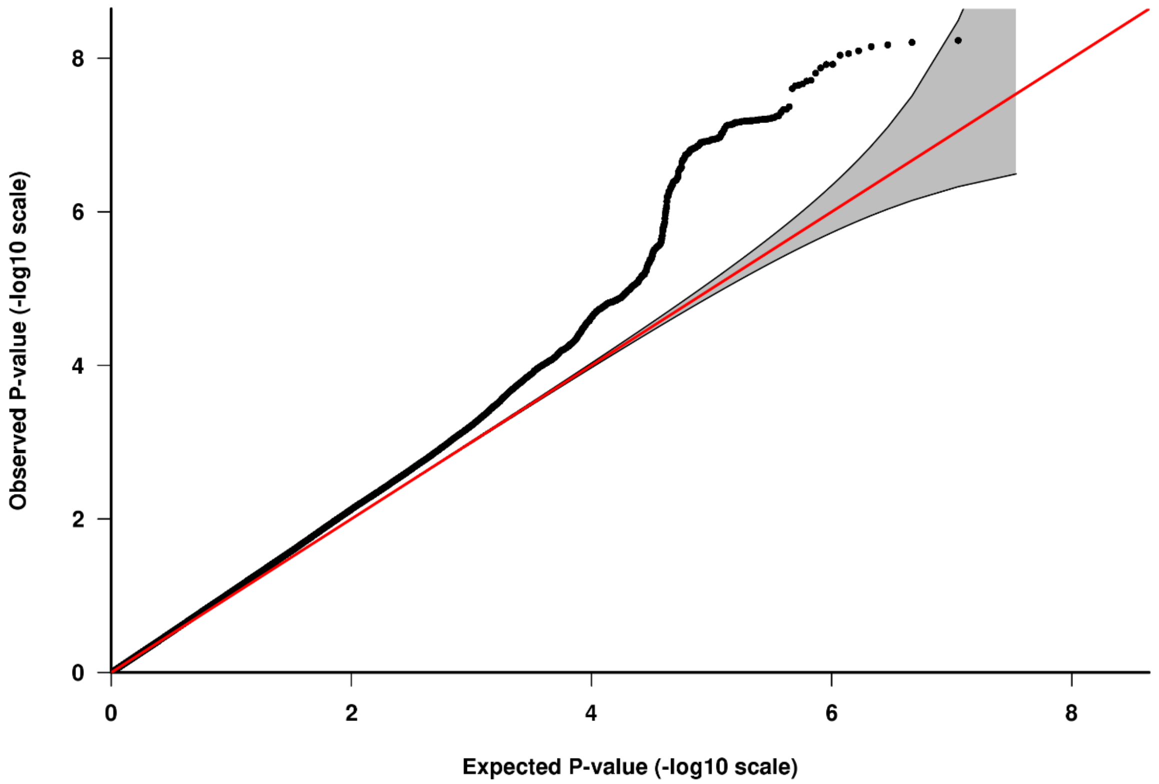

Supplementary Figure 2. Q-Q plot of the observed *P*-values for an association with probable MDD on that expected

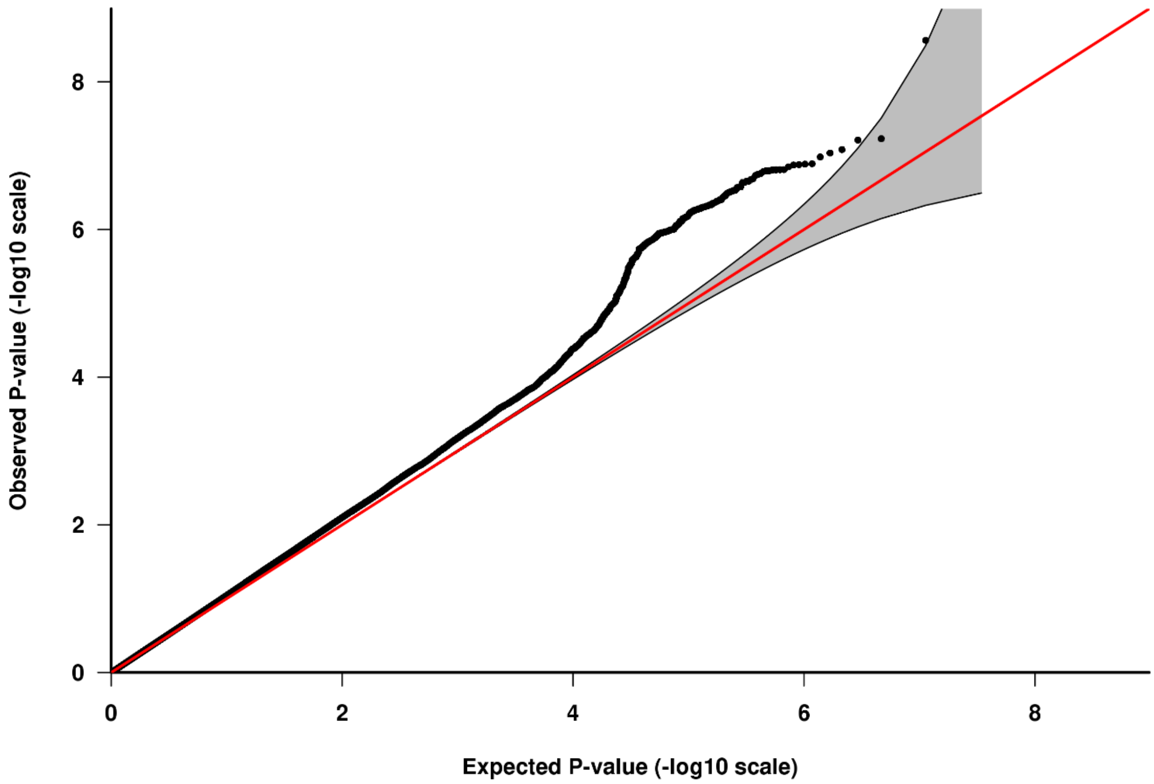

Supplementary Figure 3. Q-Q plot of the observed *P*-values for an association with ICD-coded MDD on that expected

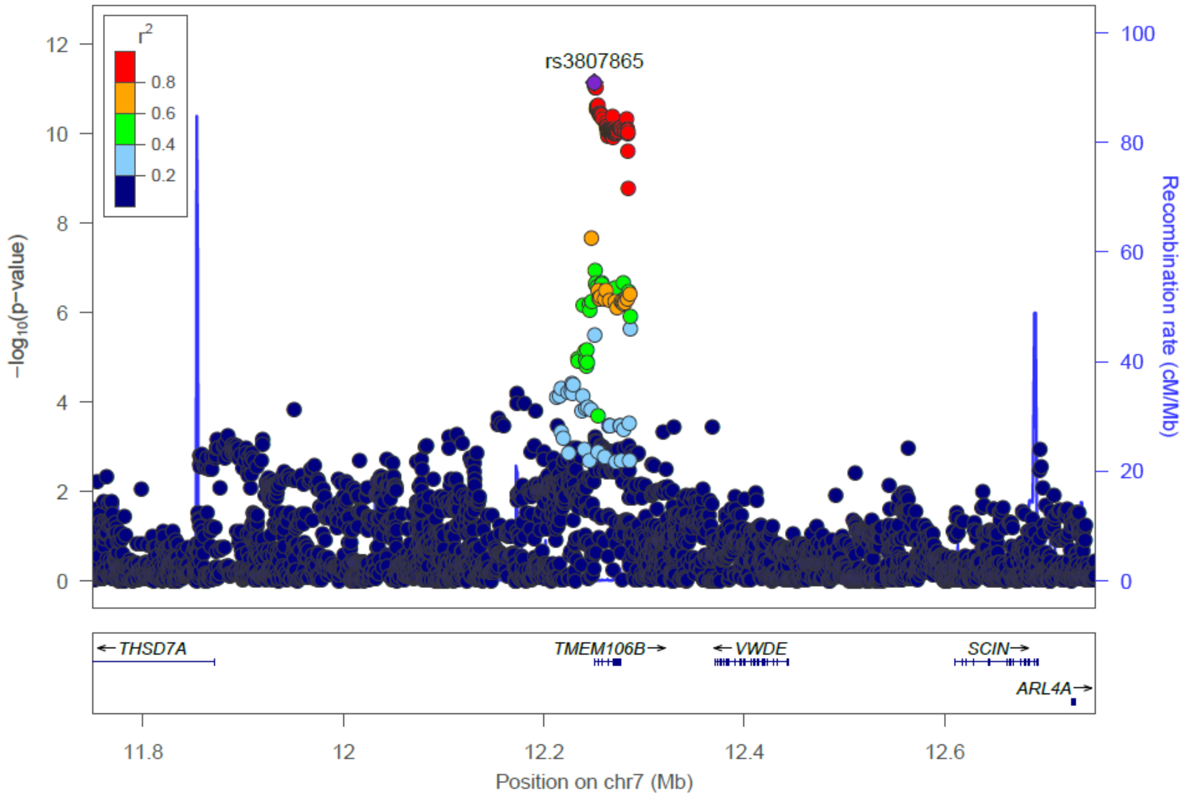

Supplementary Figure 4. Regional visualization plots centred on rs3807865 (chromosome 7, 12,250,402 bp)

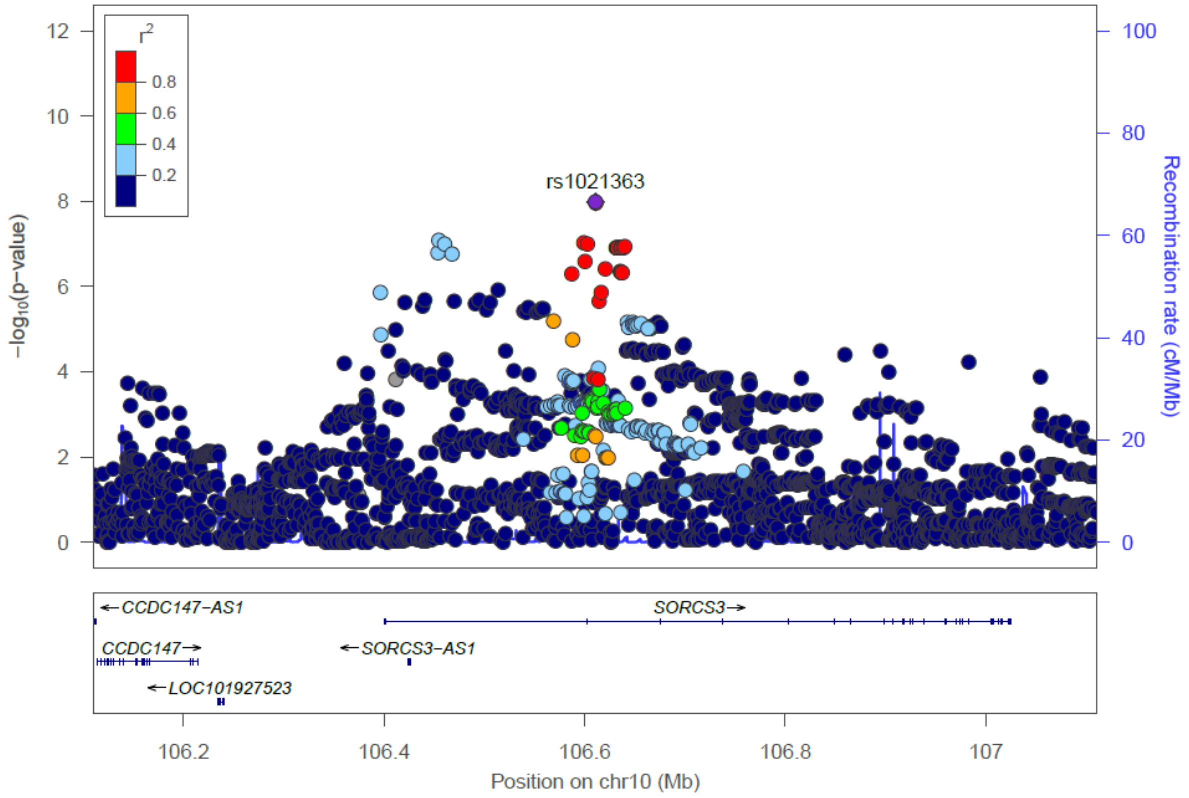

Supplementary Figure 5. Regional visualization plots centred on rs1021363 (chromosome 10, 106,610,839 bp)

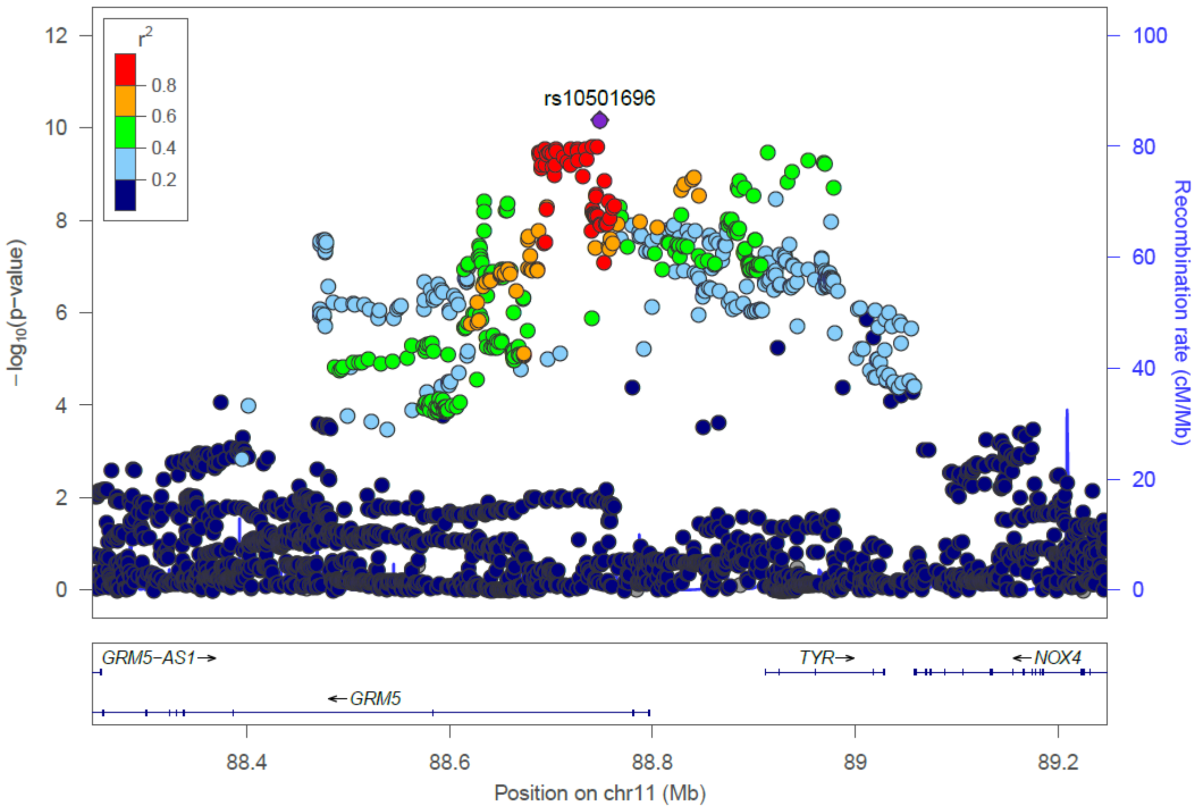

Supplementary Figure 6. Regional visualization plots centred on rs10501696 (chromosome 11 88,748,162 bp)

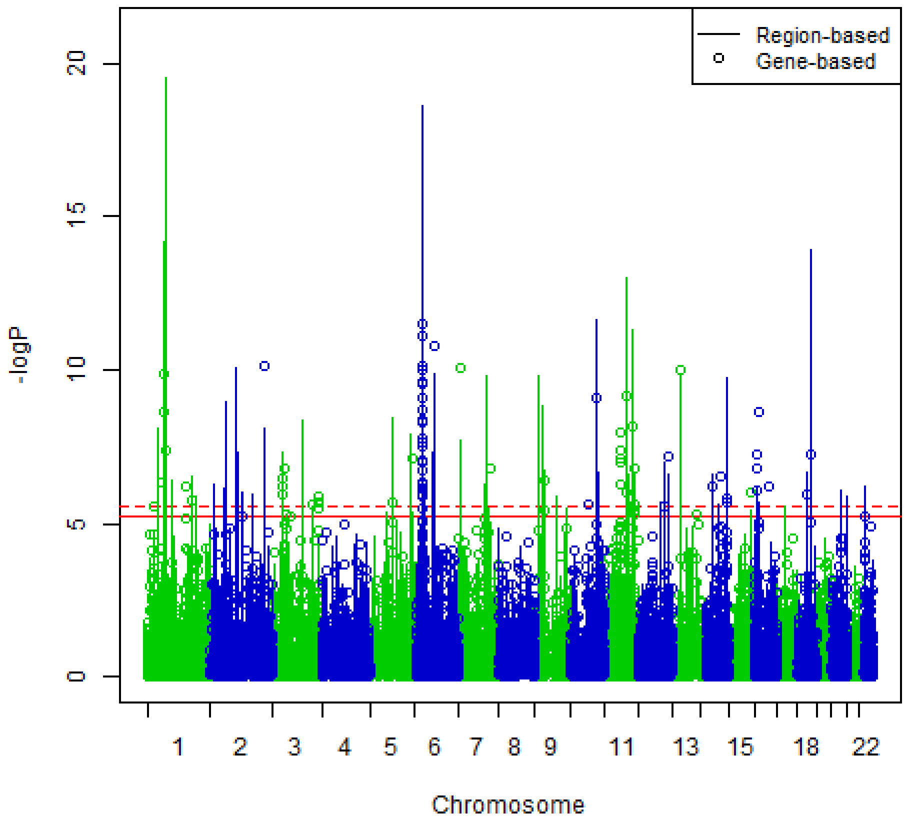

Supplementary Figure 7. Manhattan plot of the adjusted –$\log_{10}$ *P*-values of each gene or region for an association with broad depression in the UK Biobank cohort. The dotted horizontal line represents the gene-based threshold for significance and the solid horizontal line represents the region-based threshold for significance after multiple testing correction.

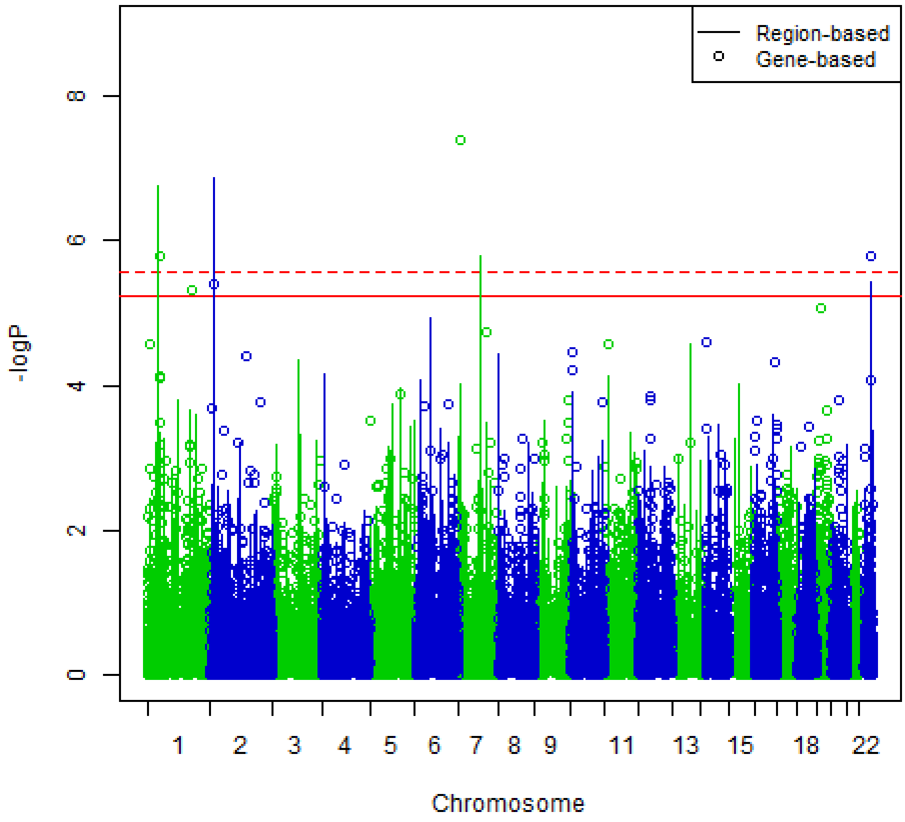

Supplementary Figure 8. Manhattan plot of the adjusted $-\log_{10}$ *P*-values of each gene or region for an association with probable MDD in the UK Biobank cohort. The dotted horizontal line represents the gene-based threshold for significance and the solid horizontal line represents the region-based threshold for significance after multiple testing correction.

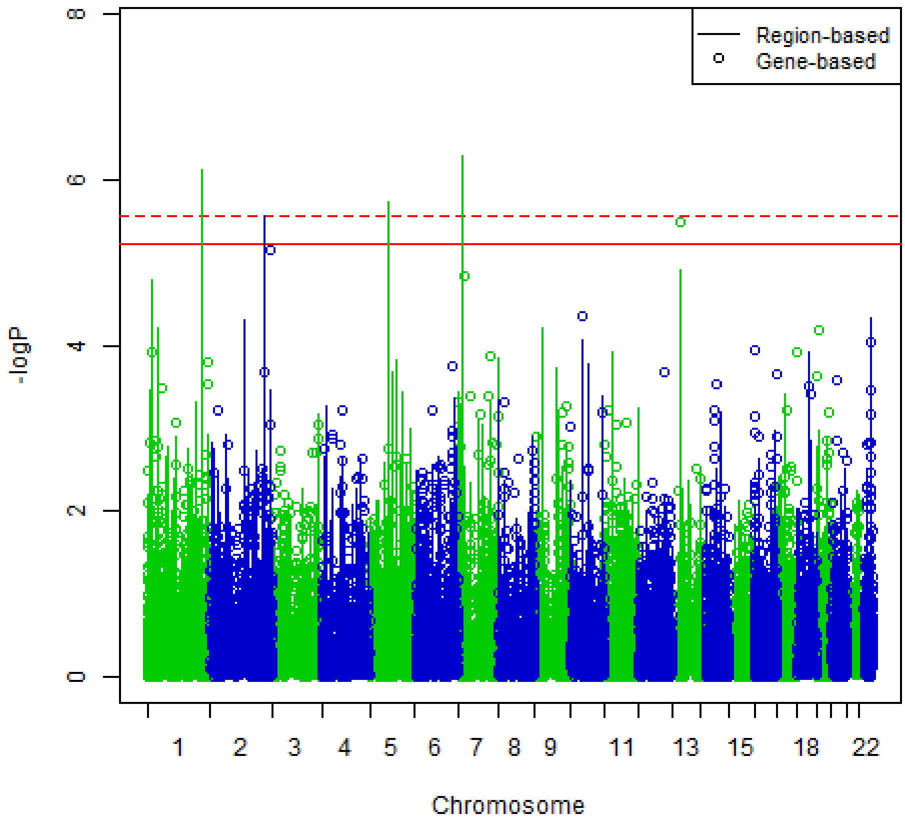

Supplementary Figure 9. Manhattan plot of the adjusted $-\log_{10}$ *P*-values of each gene or region for an association with ICD-coded MDD in the UK Biobank cohort. The dotted horizontal line represents the gene-based threshold for significance and the solid horizontal line represents the region-based threshold for significance after multiple testing correction.

Supplementary Table 1. Number of individuals (N), number of each sex, mean age in years, age range in years for each of the assessed UK Biobank phenotypes and within the respective case and control groups

Phenotype	Status	N	Males	Females	Mean Age (st.dev)	Age Range
Broad depression	Cases	113,769	40,477	73,292	56.5 (7.8)	39-73
	Controls	208,811	109,426	99,385	57.1 (8.1)	39-72
	Total	322,580	149,903	172,677	56.9 (8.0)	39-73
Probable MDD	Cases	30,603	11,346	19,527	56.1 (7.8)	40-70
	Controls	143,916	65,015	78,901	57.1 (7.9)	39-73
	Total	174,519	76,361	98,158	56.9 (7.9)	39-73
ICD-coded MDD	Cases	8,276	3,098	5,178	56.5 (7.9)	40-70
	Controls	209,308	99,961	109,347	57.6 (8.0)	39-73
	Total	217,584	103,059	114,525	57.6 (8.0)	39-73

Supplementary Table 2. Estimates of the intercept, standard error and genomic inflation factor (λ_{GC}) obtained from linkage disequilibrium score regression

**Phenotype**	**Intercept**	**Standard Error**	λ_{GC}
Broad depression	1.0079	0.0078	1.3238
Probable MDD	1.0085	0.0065	1.0802
ICD-coded MDD	0.9973	0.0061	1.0802

Supplementary Table 3. Number of individuals (N) and SNP-based heritiability (h^2) of broad depression, probable major depressive disorder (MDD) and International Classification of Diseases-coded MDD within each UK Biobank recruitment centre

	**Broad phenotype**			**Probable MDD**			**ICD-coded MDD**		
**Recruitment Centre**	**N**	**h^2**	**s.e**	**N**	**h^2**	**s.e**	**N**	**h^2**	**s.e**
Barts	5745	0.000	0.132	1754	0.122	1.174	3926	0.000	0.708
Birmingham	15519	0.115	0.051	15184	0.078	0.055	9287	0.106	0.229
Bristol	29228	0.111	0.029	13948	0.115	0.083	19285	0.011	0.149
Bury	19544	0.102	0.040	8676	0.133	0.125	14170	0.261	0.150
Cardiff	12135	0.097	0.066	3363	0.000	0.581	8643	0.000	0.330
Croydon	14686	0.062	0.055	14375	0.000	0.300	8519	0.000	0.300
Edinburgh	11951	0.187	0.071	3136	1.841	1.822	7595	1.270	1.120
Glasgow	11991	0.162	0.066	3414	0.000	0.990	8663	0.000	0.630
Hounslow	14398	0.180	0.059	14128	0.242	0.072	8203	0.208	0.324
Leeds	29796	0.111	0.027	10622	0.000	0.126	21299	0.000	0.132
Liverpool	21446	0.076	0.035	16151	0.053	0.052	14456	0.134	0.120
Mancester	8693	0.120	0.089	4741	0.000	0.200	6077	0.265	0.350
Middlesborough	14188	0.107	0.054	13785	0.013	0.056	9097	0.173	0.159
Newcastle	24117	0.156	0.034	7203	0.004	0.064	18910	0.080	0.118
Nottingham	23104	0.193	0.036	9474	0.048	0.125	16083	0.269	0.164
Oxford	9631	0.233	0.086	2503	1.000	0.827	6445	0.691	0.505
Reading	20798	0.189	0.043	5301	0.380	0.462	14071	0.184	0.273
Sheffield	20558	0.129	0.034	19892	0.139	0.044	12409	0.000	0.175
Stockport	301	1.560	2.560	291	1.810	3.162	177	5.150	16.610
Stoke	12682	0.000	0.060	4584	0.095	0.274	9050	0.000	0.263
Swansea	1586	0.575	0.466	1525	0.466	0.511	929	2.368	3.386
Wrexham	483	0.000	1.513	469	1.660	1.590	290	3.750	6.340

Supplementary Table 4. Recruitment centres, number of individuals (N) and SNP-based heritiability (h^2) and standard error (s.e.) of broad depression, probable major depressive disorder (MDD) and International Classification of Diseases-coded MDD within each geographical region

		Broad phenotype			Probable MDD			ICD-coded MDD		
**Region**	**Recruitment centres in region**	**N**	**h^2**	**s.e**	**N**	**h^2**	**s.e**	**N**	**h^2**	**s.e**
London	Barts, Croydon, Hounslow	34829	0.146	0.025	30257	0.111	0.034	20648	0.079	0.128
South	Bristol, Oxford, Reading	59657	0.145	0.015	21752	0.247	0.067	39801	0.254	0.085
Wales	Cardiff, Swansea, Wrexham	14204	0.084	0.054	5357	0.275	0.225	9862	0.000	0.287
Midlands	Birmingham, Nottingham, Stoke	51305	0.130	0.017	29242	0.067	0.035	34420	0.144	0.072
Northwest	Bury, Liverpool, Manchester, Stockport	49984	0.123	0.017	29859	0.045	0.032	34880	0.167	0.058
Northeast	Leeds, Middlesborough, Newcastle, Sheffield	88659	0.122	0.010	51502	0.087	0.020	61715	0.096	0.037
Scotland	Edinburgh, Glasgow	23942	0.178	0.035	6550	0.000	0.641	16258	0.000	0.402

Supplementary Table 5. The genetic correlations (r_g), standard errors (s.e.) and *P*-values (testing the hypothesis that $r_g = 0$) and the *P*-value of $r_g = 1$ (testing the hypothesis that $r_g = 1$) between each UK Biobank phenotype

**Phenotype**	**r_g with broad depression (s.e.)**	***P*-value of $r_g = 0$ with broad depression**	**P-value of $r_g = 1$ with broad depression**	**r_g with probable MDD (s.e.)**	***P*-value of $r_g = 0$ with probable MDD**	***P*-value of $r_g = 1$ with probable MDD**
Broad depression	-	-	-	0.871 (0.050)	4.18×10^{-67}	0.0098
Probable MDD	0.871 (0.050)	4.18×10^{-67}	0.0098	-	-	-
ICD-coded MDD	0.863 (0.046)	5.03×10^{-80}	0.0029	0.848 (0.052)	4.21×10^{-59}	0.0035

Supplementary Table 6. Overlap of genes within the gene-sets associated ($P_{corrected} < 0.05$) with broad depression

		Gene set				
**Number of genes in gene-set**	**Gene set**	GO_EXCITATORY_SYNAPSE	GO_MECHANOSENSORY_BEHAVIOR	GO_POSTSYNAPSE	GO_NEURON_SPINE	GO_DENDRITE
184	GO_EXCITATORY_SYNAPSE	-	0.250	0.973	0.452	0.516
12	GO_MECHANOSENSORY_BEHAVIOR	3	-	0.333	0.250	0.417
354	GO_POSTSYNAPSE	179	4	-	0.991	0.508
115	GO_NEURON_SPINE	52	3	114	-	0.991
425	GO_DENDRITE	95	5	180	114	-

Values on the lower diagonal are the number of overlapping genes between gene sets. Values on the upper diagonal are the proportion of overlapping genes within the gene set containing the lower number of genes

Supplementary Table 7. Cross tabulation of case, control, and not available (na) status for each pair of UK Biobank phenotypes

		Broad Depression		
		**Cases**	**Controls**	**na**
**Probable MDD**	**Case**	30603	0	0
	**Control**	66176	77598	142
	**na**	16990	131213	8650

		Broad Depression		
		**Cases**	**Controls**	**na**
**ICD-coded MDD**	**Case**	8276	0	0
	**Control**	53491	155469	348
	**na**	52002	53342	8444

		Probable MDD		
		**Cases**	**Controls**	**na**
**ICD-coded MDD**	**Case**	8276	0	0
	**Control**	0	111109	98199
	**na**	22327	32807	58654
